# Semantic Signature: Comparative Interpretation of Gene Expression on a Semantic Space

**DOI:** 10.1155/2016/5174503

**Published:** 2016-05-03

**Authors:** Jihun Kim, Keewon Kim, Ju Han Kim

**Affiliations:** ^1^Seoul National University Biomedical Informatics (SNUBI), Seoul 110-799, Republic of Korea; ^2^LabGenomics Clinical Research Institute, LabGenomics, Seongnam 463-400, Republic of Korea; ^3^Department of Rehabilitation Medicine, Seoul National University College of Medicine, Seoul 110-799, Republic of Korea; ^4^Departments of Biomedical Engineering, Seoul National University College of Medicine, Seoul 110-799, Republic of Korea; ^5^Systems Biomedical Informatics Research Center, Division of Biomedical Informatics, Seoul National University College of Medicine, Seoul 110-799, Republic of Korea

## Abstract

*Background*. Interpretation of microarray data remains challenging because biological meaning should be extracted from enormous numeric matrices and be presented explicitly. Moreover, huge public repositories of microarray dataset are ready to be exploited for comparative analysis. This study aimed to provide a platform where essential implication of a microarray experiment could be visually expressed and various microarray datasets could be intuitively compared.* Results*. On the semantic space, gene sets from Molecular Signature Database (MSigDB) were plotted as landmarks and their relative distances were calculated by Lin's semantic similarity measure. By formal concept analysis, a microarray dataset was transformed into a concept lattice with gene clusters as objects and Gene Ontology terms as attributes. Concepts of a lattice were located on the semantic space reflecting semantic distance from landmarks and edges between concepts were drawn; consequently, a specific geographic pattern could be observed from a microarray dataset. We termed a distinctive geography shared by microarray datasets of the same category as “semantic signature.”* Conclusions*. “Semantic space,” a map of biological entities, could serve as a universal platform for comparative microarray analysis. When microarray data were displayed on the semantic space as concept lattices, “semantic signature,” characteristic geography for a microarray experiment, could be discovered.

## 1. Background

Microarray experiments provide high-throughput gene expression profiles to address biological questions for a specific condition. It has been challenging so far to extract biological implication from huge matrices of numeric data and to represent it in a concise and intuitive manner. Also, as microarray datasets accumulate in public repositories, it has become another important issue in microarray analysis how to compare multiple datasets and integrate them [1].

As for the first issue (analysis of an individual microarray experiment), semantic approach was suggested as one of main strategies. For example, clusters of genes with similar expression profiles could be assigned with Gene Ontology (GO) terms or related pathways [2]. However, most of clustering analyses with ontological annotation merely provided long list of biological terms for clusters; they failed to represent functional relationship between clusters and the whole picture of microarray experiments could hardly be grasped. Concept lattice analysis, or formal concept analysis, was proposed as a way to summarize biological information from clusters without annotation redundancy [3]. Concept lattice analysis is a mathematical technique that recognizes hierarchical structure from a relation matrix of objects (clusters) and attributes (annotations) and represents it as a graph (lattice). In this way, clusters are depicted as nodes and set-inclusion relationships of their annotations are drawn as edges in a concept lattice, which can be viewed as an executive summary of the microarray data.

The second issue (comparative microarray analysis) is more elusive. External datasets of various conditions can be compared to interpret one microarray experiment of interest [4–9]. Or multiple experiments can be gathered and analyzed to deduce causality or association between genes [10–20]. Such comparative analyses can reduce noises that individual experiments might contain and can help with arranging vast datasets to reveal novel relationship between phenotypes or gene expressions. However, comparisons are inevitably dependent on reference data being compared and platforms on which comparisons are made. In particular, scope of reference datasets can be restricted if the platform of comparative analysis is not compatible with diverse microarray experiments.

The current study was motivated to provide a universal, not being influenced by formats of data and platform where microarray data could be visually presented and compared. Furthermore, we hoped it could convey biological implication of experiments. For that purpose, we constructed “semantic space,” a map of biological entities. Just as a map encompasses the whole territory of interest, the semantic space employed gene sets of Molecular Signature Database (MSigDB) as landmarks. MSigDB was a collection of gene sets from various sources (positional, curated, motifs, computational, and GO gene sets) and could be regarded as a representation of the biological world at this time point [6]. The coordinates of those landmarks were determined by semantic distances between them based on a predefined semantic similarity measure [21].

On the semantic space, microarray data were mapped as a concept lattice. A concept lattices was produced by formal concept analysis, with clusters of similar expression profiles as objects and GO annotations as attributes. Each cluster was located on the semantic space considering its semantic distance from nearest landmarks and edges of the lattice between clusters were also drawn.

**Figure 1:**
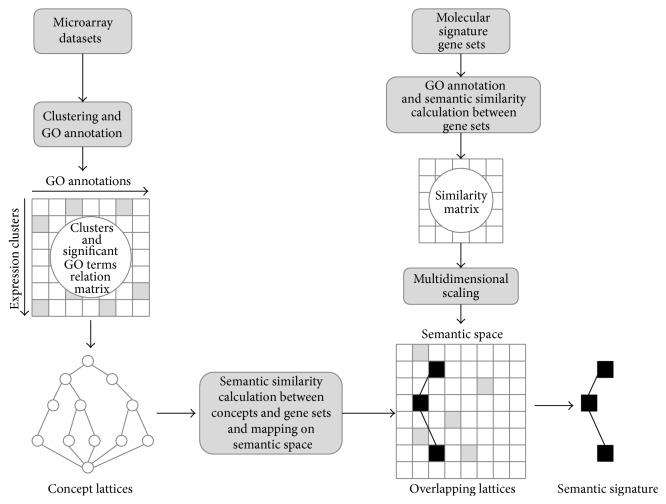
Schematic diagram of the study. Microarray experiments are transformed into concept lattices as illustrated on the left panel of the figure. Semantic space is constructed based on semantic similarities between gene sets as illustrated on the right panel. Concept lattices are then superimposed on the semantic space to reveal a semantic signature and shared geography by homogeneous experiments.

A concept lattice described on the semantic space makes a distinctive topography on the semantic space, termed as a “semantic signature.” Semantic signature would give information of how gene clusters of a microarray dataset were related to biological landmarks at a glance. Furthermore, we compared various microarray datasets by simply overlapping their semantic signatures on the semantic space. And we tested whether data of similar experiment paradigm resulted in similar semantic signatures and whether those of different paradigm did different semantic signatures.  Figure 1 illustrates the brief process for construction of semantic space and plotting of semantic signature.

## 2. Methods

### 2.1. Construction of Semantic Space

Gene sets of MSigDB (http://www.broad.mit.edu/gsea/msigdb) imported to generated landmarks of semantic space. MSigDB collected gene sets from various sources and organized them into 5 categories: positional, curated, motifs, computational, and GO gene sets [6]. In this study, we adopted all the gene sets from mouse species, registered in GO biological process: 192 gene sets in total.

Semantic distances among the gene sets were determined as follows. First, we annotated the gene sets significant GO terms; statistical significance was determined by hypergeometric probability with Bonferroni correction [3]. Then, distance (sim) between two gene sets (*P*_*i*_, *P*_*j*_) was calculated by best-match-average (bma) of term-to-term distances from each gene set [34]:

(1) $$\begin{array}{c} \underset{bma\left(P_{i},P_{j}\right)}{sim}=\frac{{\mathrm{avg}}_{t1\in P_{i}}\left(\max_{t2\in P_{j}}\left({sim}_{lin\left(t1,t2\right)}\right)\right)+{\mathrm{avg}}_{t2\in P_{j}}\left(\max_{t1\in P_{i}}\left({sim}_{lin\left(t1,t2\right)}\right)\right)}{2}. \end{array}$$

Distance between two terms (*t*1, *t*2) was computed using Lin's semantic similarity measure [35]. Lin's measure quantified “information content” of a term, by enumerating frequency of the genes that were annotated with the term or its descendant terms:

(2) $$\begin{array}{c} freq\left(\;t\;\right)=genes\left(\;t\;\right)+\underset{x\in descendant\left(t\right)}{\sum }freq\left(\;x\;\right). \end{array}$$

Lin's semantic similarity measure (sim_lin) was then determined as follows:

(3) simlint1,t2=maxt∈St1,t2⁡2·log⁡ptlog⁡pt1+log⁡pt2,where  pt=freqtfreqroot.

All pair-wise distances of the gene sets were computed and summarized in a matrix. Afterwards, multidimensional scaling was applied to generate 2D coordinates of the gene sets as landmarks in semantic space (cmdscale package, R, https://www.r-project.org/). Finally, the landmarks were plotted in a plane using scalable vector graphics and finally the semantic space was constructed.

### 2.2. Concept Lattices from Microarray Datasets

From one microarray dataset, one concept lattice was generated as follows. First, *k*-means clustering analysis produced 100 clusters for a dataset. Then, clusters were annotated with significant GO terms based on hypergeometric probability with Bonferroni correction. Third, a relation matrix of clusters and annotations was built. Fourth, a formal concept analysis transformed the relation matrix into a concept lattice with clusters as objects (extent) and GO terms as attributes (intent). For graphic representation, this study adopted Ganter's algorithm [36]. Accordingly, a concept was a set of clusters sharing GO terms. Edges between concepts in the lattice indicated set-inclusion relationship. Therefore, the graph structure of a concept lattice could convey the whole information of a relation matrix of clusters and GO annotations. (http://www.snubi.org/software/biolattice/.)

### 2.3. Discovery of Semantic Signature

**Figure 2:**
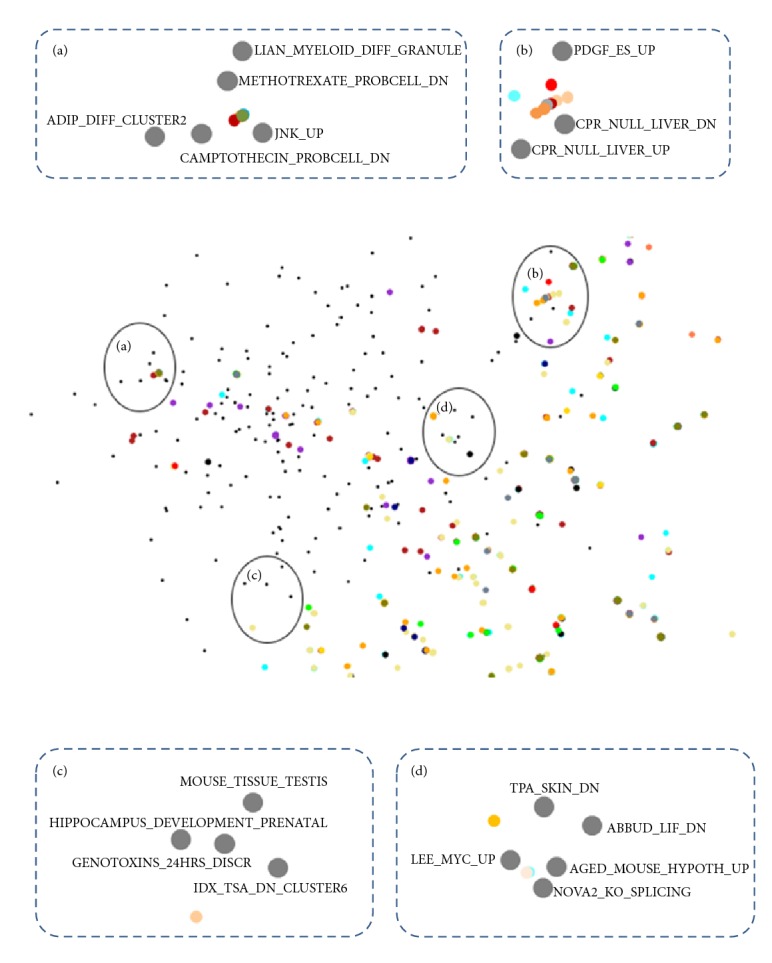
The semantic space. (a) The whole semantic space. (b) Magnified view of specific regions of the semantic space. On magnified views, title of each landmark gene set is presented near it. (a)* LIAN_MYELOID_DIFF_GRANULE*: granule constituents expressed during mouse promyelocytic cell line cell differentiation;* METHOTREXATE_PROBCELL_DN*: downregulated in pro B cells FL5 12 following treatment with methotrexate;* JNK_UP*: upregulated by expression of constitutively active JNK in 3T3 cells;* CAMPTOTHECIN_PROBCELL_DN*: downregulated in pro B cells FL5 12 following treatment with camptothecin;* ADIP_DIFF_CLUSTER2*: strongly upregulated at 2 hours during differentiation of 3T3 L1 fibroblasts into adipocytes cluster 2. (b)* PDGF_ES_UP*: upregulated by PDGF in mouse embryonic stem cells via microarray coupled gene trap mutagenesis;* CPR_NULL_LIVER_DN*: downregulated in mouse liver tissue from mice in which NADPH cytochrome P450 reductase CPR was specifically deleted in the liver by cre lox recombination versus lox only controls;* CPR_NULL_LIVER_UP*: upregulated in mouse liver tissue from mice in which NADPH cytochrome P450 reductase CPR was specifically deleted in the liver by cre lox recombination versus lox only controls. (c)* MOUSE_TISSUE_TESTIS*: genes expressed specifically in mouse testis tissue;* HIPPOCAMPUS_DEVELOPMENT_PRENATAL*: highly expressed in prenatal mouse hippocampus cluster 1;* GENOTOXINS_24HRS_DISCR*: group of genes whose regulation pattern significantly discriminates between direct cisplatin methyl methanesulfonate mitomycin C and indirect taxol hydroxyurea etoposide genotoxins 24 hours following treatment of mouse lymphocytes TK 3 7 2C;* IDX_TSA_DN_CLUSTER6*: strongly downregulated at 2 hours during differentiation of 3T3 L1 fibroblasts into adipocytes with IDX insulin dexamethasone and isobutyl xanthine versus fibroblasts treated with IDX TSA to prevent differentiation cluster 6. (d)* TPA_SKIN_DN*: downregulated in murine dorsal skin cells 6 hours after treatment with the phorbol ester carcinogen TPA;* ABBUD_LIF_DN*: genes downregulated by LIF treatment 10 ng mL overnight in AtT20 cells;* LEE_MYC_UP*: genes upregulated in hepatoma tissue of Myc transgenic mice;* AGED_MOUSE_HYPOTH_UP*: upregulated in the hypothalamus of BALB c mice aged 22 months compared to young 2-month controls;* NOVA2_KO_SPLICING*: genes that are alternatively spliced in the neocortex of mice deficient in the neuron specific splicing factor Nova2 compared to wild type controls.

Once semantic space was constructed and a concept lattice of microarray experiments was generated, the concept lattice could be mapped on the semantic space. Because extents of a concept lattice were clusters, or sets of genes, semantic distance from the concept to any landmark on the semantic space could be calculated just as distances between landmark gene sets were calculated (see Figure 2). To appropriately place the concept on the semantic space, 3 nearest landmarks were found for each concept. The coordinate of the concept was then determined within the triangle of the 3 nearest landmarks according to relative distances to the 3 vertices. In this way, all the concepts of a concept lattice could be located on the semantic space. Edges between concepts were drawn as well. Finally, the whole concept lattice of microarray experiments was depicted on the semantic space and unique geographic feature could be observed (Figure 2).

In order to compare multiple microarray datasets, concept lattices of them were mapped on the semantic space simultaneously. A common geographic pattern of homogeneous microarray datasets on the semantic space, termed as “semantic signature,” was derived by investigating overlapping or closely neighbouring concepts and edges. To visualize the semantic signature, overlapped edges were emphasized by increasing colour intensity according to the overlapping frequency.

### 2.4. Microarray Datasets

Microarray data of hepatotoxic agent experiments was obtained from a toxicogenomics study by Toxicogenomics Research Center (TGRC). The study applied 12 toxic agents to mice orally or intraperitoneally and observed gene expression profiles from liver specimen according to time course and dosage. Twelve toxic agents were D-galactosamine, ethanol, tetracycline, valproic acid, methotrexate, ANIT, methylenedianiline, phenytoin, thiabendazole, 6-mercaptopurine, phenylbutazone, and diclofenac.

Twenty microarray datasets were downloaded from GEO. Datasets were selected among mouse (*Mus musculus*) datasets if they included sufficient number of conditions (8 or more) for clustering analysis and experimental condition and tissues were explicitly described. The datasets were categorized per condition and tissue: toxin-related (GDS322, GDS2043), development-related (half of GDS2577, GDS2227, GDS2398, GDS2521, GDS1695, GDS568, GDS2202, GDS2203, GDS2098, and GDS2743), and cancer-related (half of GDS2577, GDS1110, GDS604, GDS2640, and GDS2554) conditions; neural (GDS2227, GDS1110, GDS604, GDS887, GDS2850, and GDS2159), hematopoietic (GDS322, GDS2398, GDS2521, GDS1695, GDS568, GDS2640, and GDS2554), and germinal (GDS2043, GDS2202, GDS2203, and GDS2098) tissues.

## 3. Results

### 3.1. Semantic Space

“Semantic space” is shown in Figure 2. 192 gene sets from MSigDB are marked on it as its landmarks. Figure 2 shows the whole picture of the semantic space. Each vertex indicates one gene set. In this study, the semantic space was constructed using scalable vector graphics (SVG) so that the map could be magnified without being blurred. Magnified views of specific regions are illustrated in Figure 2, (a)~(d). Given that locations of those gene sets were determined according to semantic distances between them, gene sets sharing similar GO annotations congregated in close vicinity. For example, most of gene sets in the upper right circle of Figure 2(b) were related mostly to adipose or secretary cell development.

### 3.2. Semantic Signature: An Example of Hepatotoxic Agent Experiment

As an exemplary case, we assessed the semantic signature of a microarray dataset from hepatotoxic agent experiment (see Section 2). The experiment observed acute hepatotoxic effect of 12 toxicants in mouse after oral or intraperitoneal injection and produced 12 microarray data. Expression profile from each agent was converted into a concept lattice and then mapped on the semantic space. Obtained figures of 12 concept lattices were not identical but their concepts and edges were overlapping or closely neighbouring on the semantic space. Those common edges were visually emphasized by grading the colour intensity in proportion to overlapping frequency (Figure 2). The common concepts and edges were clustered along the right border of the semantic space making figure of a saw tooth, which was then determined as “semantic signature” of the hepatotoxicity experiment.

### 3.3. Semantic Signatures: Comparison of Heterogeneous Experiments

**Figure 3:**
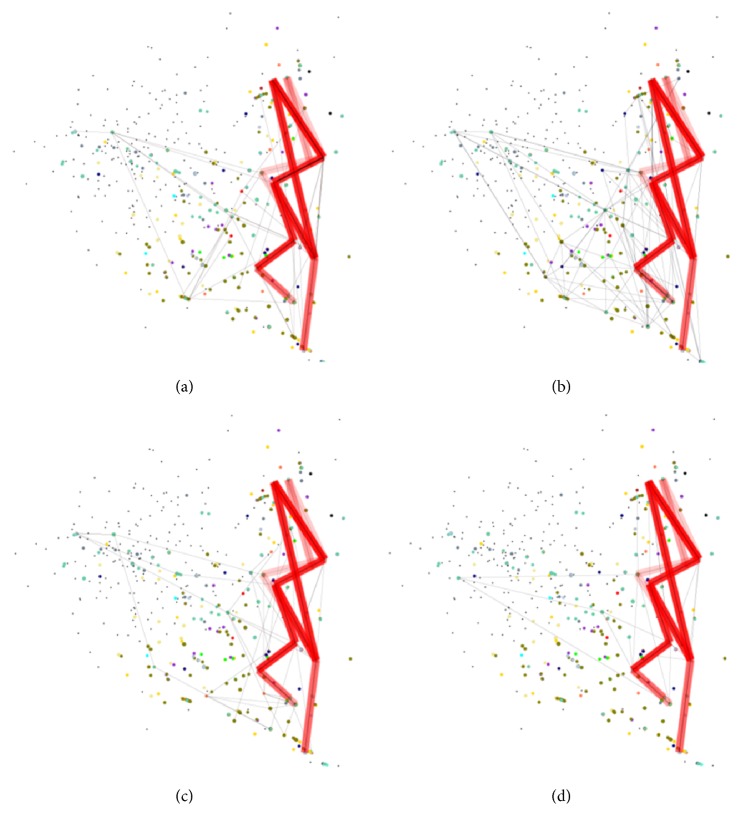
Semantic signature of hepatotoxic agent experiments. Concept lattice of 4 exemplary microarray experiments are represented on the semantic space: (a) tetracycline, (b) ethanol, (c) methylenedianiline, and (d) phenytoin. Shared edges are expressed in bold red lines with colour intensity proportional to overlapping frequency. The common features denote the semantic signature of hepatotoxic agent experiments.

Other various datasets from different experimental conditions or from different tissues were represented on the semantic space. Twenty heterogeneous microarray datasets were obtained from GEO (Gene Expression Omnibus, http://www.ncbi.nlm.nih.gov/geo/) and they were categorized into 3 conditions (toxin-related, cancer-related, and development-related experiments) and 3 tissues (germinal, hematopoietic, and neural tissues) based on descriptions provided by GEO. To discover semantic signatures per condition or tissue, common concepts and edges were depicted in the same way described above (Figure 3). The obtained semantic signatures exhibited distinctive patterns for each experiment category. Each SVG is available in

 
http://www.snubi.org/software/biolattice/bioclass/canceroverlap.htm;
 
http://www.snubi.org/software/biolattice/bioclass/devoverlap.htm;
 
http://www.snubi.org/software/biolattice/bioclass/germoverlap.htm;
 
http://www.snubi.org/software/biolattice/bioclass/hemaoverlap.htm;
 
http://www.snubi.org/software/biolattice/bioclass/neuroverlap.htm;
 
http://www.snubi.org/software/biolattice/bioclass/toxoverlap.htm.

### 3.4. Validation of Semantic Signature: Semantic Distance among Concept Lattices

**Figure 4:**
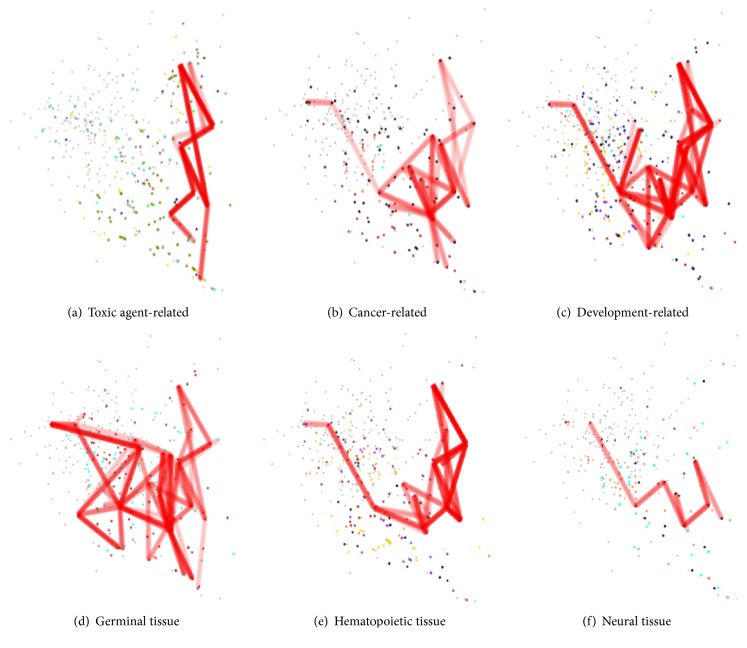
Semantic signatures of heterogeneous experiments. Various microarray datasets are categorized per condition or tissue and their semantic signatures are presented in red lines. The conditions are (a) toxic agent-related, (b) cancer-related, and (c) development-related experiments. The specimens are from (d) germinal, (e) hematopoietic, and (f) neural tissues.

**Figure 5:**
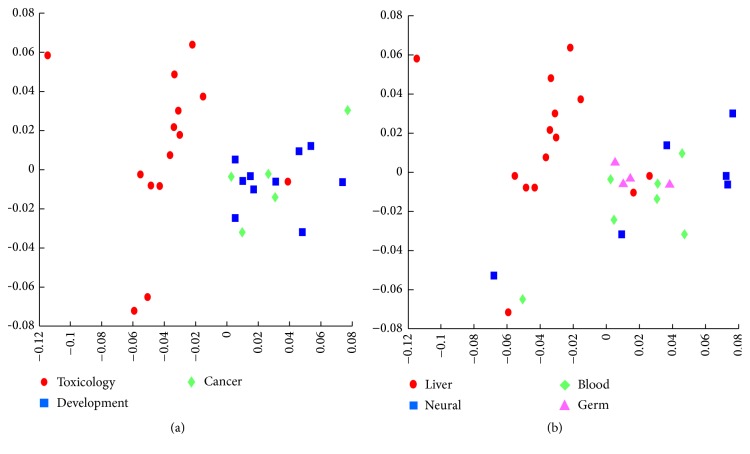
Semantic distance among concept lattices. Concept lattices are plotted on 2D space according to semantic distance between them. Concept lattices of the same (a) experimental condition or (b) tissues are located closely.

As shown in Figures 4 and 5, it might be asserted that geographic patterns from homogeneous experiments looked alike and those from heterogeneous experiments looked different in the semantic space. However, such visual judgement of similarity remained somewhat subjective and arbitrary without quantitative validation. To circumvent the problem, we tested whether concept lattices of the same experiment category were closer than those of different category, based on semantic similarity among concept lattices. The calculation of the semantic distances between two lattices followed the same strategy that was used for construction of the semantic space (Figure 4). Concept lattices of the same experiment conditions were closely located (Figure 4(a)). And the lattices of development-related experiments were closer to the lattices of cancer-related experiments than to those of toxic agent-related ones as was indicated by topographic similarity between their semantic signatures. Also, concept lattices of the same tissue were located in closer vicinity (Figure 4(b)).

## 4. Discussion

### 4.1. Biological Interpretation of Semantic Signatures I

For biological interpretation of the semantic signature from the hepatotoxic agent experiments, 8 most frequently neighboured landmark gene sets in the signature were listed (Table 1). One of the landmarks was a gene set that was upregulated by transcription factor Hxc-8, which was known to interact with hematopoietic activities in the liver tissue, suggesting that reactive hematopoiesis is induced by the hepatotoxic agents (LEI_HOXC8_UP gene set) [22]. Another landmark gene set was YEN MYC MUT which was generated from myc mutation mouse; phenobarbital (a hepatotoxic agent included in this experiment) was known to induce apoptosis and carcinogenesis of hepatocytes via c-myc expression [23]. Other landmarks included a gene set which was associated with inflammatory change induced by TNF (TNFALPHA_ADIP_UP) [24] and gene sets related to apoptotic of inflammatory signalling by insulin receptor (ROS_MOUSE_AORTA_UP and IGFR_IR_UP) [25]. While the semantic signatures of hepatotoxic agent experiments shared common landmarks with each other as described above (thick red line in Figure 3), each concept lattice from different hepatotoxic agent also exhibited different patterns (thin red line in Figure 3). For example, concept lattice from ethanol treatment showed more nodes and edges than that from phenytoin, which could be interpreted as ethanol resulting in perturbation in more gene clusters than phenytoin. The finding was consistent with general biological knowledge that ethanol is more toxic than phenytoin to the liver.

### 4.2. Biological Interpretation of Semantic Signatures II

When geography of the semantic signatures of various microarray datasets was compared, the semantic signature from cancer-related experiments was more similar to that from development-related experiments than to that from toxic agent-related ones. The finding was consistent with an established biological knowledge that one of main oncogenic mechanisms was related to uninhibited activation of developmental pathways. Specifically, the semantic signature of development-related dataset was closely neighboured by landmarks that included differentially expressed genes during cell differentiation (LIAN_MYELOID_DIFF_GRANULE, HIPPOCAMPUS_DEVELOPMENT_NEONATAL, and PARK_HSC_VS_MPP_UP) and landmarks that were downregulated genes by immune-modifying or antineoplastic drugs (METHOTREXATE_PROBCELL_DN and CANCERDRUGS_PROBCELL_DN). The semantic signature of cancer-related experiments contained landmark gene sets associated with oncogene activation (LEI_HOXC8_UP and YU_CMYC_UP), oncogene mutation (YEN_MYC_MUT), antineoplastic agents (CANCERDRUGS_PROBCELL_DN and GENOTOXINS_ALL_4HRS_REG), and cell differentiation (LIAN_MYELOID_DIFF_GRANULE and LIAN_MYELOID_DIFF_RECEPTORS).

### 4.3. Representation of Semantic Space on 2D Space

This study was not the first that attempted to graphically represent biological entities. Several studies have provided “map” of diverse biological objects, such as genes or proteins, based on ontological annotations, structural similarity, or sequence similarities [26–28]. However, those studies employed their map only to illustrate specific study results. To the authors' knowledge, semantic space was the first that developed a comprehensive map of biological entities as a reference frame where heterogeneous experiments could be represented and compared.

In this study, we constructed semantic space on 2D space for convenience purpose. But positional relationship of components, especially if they were numerous, should be distorted when represented on lower dimension. A previous work of “yeast functional map” evaluated deforming stress from multidimensional scaling as the dimensionality was changed [26]. It reported 5D space as optimal and that increasing the dimensionality from 2D to 3D did not yield satisfactory decrease of stress, suggesting that 2D space could be a reasonable choice.

### 4.4. Measures for Semantic Distance

The current study adopted Lin's semantic similarity measure for the calculation of term-to-term semantic distance and best-match-average of it for the calculation of set-to-set distances [29]. Other various measures for semantic distance could be considered, such as Resnik's [30], Jiang and Conrath's [31], or Wang et al.'s measure [32] instead of Lin's measure. Similarly, best-match-average method could be replaced by simple average, maximum, or maximum weighted by information content for set-to-set calculation. Jaccard distance could also be employed, which did not require calculation of term-to-term distances [33].

To determine the optimal measure for semantic distance, we evaluated distribution of semantic distances between gene sets by different combination of measures. As a result, best-match-average of Lin's measure showed the largest variation of the distance values. Thus, it was chosen for the current study because widely distributed distances reduced distortion stress during dimension reduction. In addition, we also assessed how close were the 3 nearest landmarks that determined a coordinate of each concept in the semantic space, by calculating area of the triangle composed of the 3 landmarks. It was assumed that if 3 nearest landmarks were widely dispersed, placing the concept within the triangle would give less information for biological interpretation. The combination of Lin's measure and best-match-average method resulted in tolerable size of triangles (data not shown).

### 4.5. Limitations and Future Study

A few limitations should be noted in this study. As already mentioned, 2D representation of numerous landmarks could not avoid inaccuracy in their location. Another limitation is the fact that it could not be claimed that 192 gene sets from MSigDB represented all necessary spots of biological world and the semantic space spanned sufficiently wide territory. Lastly, determination of semantic signature included arbitrary component. Although visual representation of numeric data could help intuitive interpretation, similarity versus difference of geographic pattern should include subjective judgement inevitably.

Future research should consider expression of semantic space in higher dimension and could expand semantic space by merging other sources of biological data. In addition, we should think over to develop a quantitative and objective way by which similarity/difference of semantic signatures could be assessed.

## 5. Conclusions

Semantic space was constructed as a map of biological entities based on their relative semantic distances. When concept lattices were projected on the semantic space, “semantic signature,” which was defined as specific geographic patterns observed on it, allowed intuitive interpretation of microarray experiments. Comparison of semantic signatures of various microarray datasets revealed distinctive features according to experiment conditions or tissues. In conclusion, “semantic space” could serve as a universal platform for comparative microarray analysis and “semantic signature” could be discovered.

## Figures and Tables

**Table 1:** Landmarks within semantic signature of hepatotoxic agent experiment. Eight most frequently overlapped gene sets among concepts lattices of hepatotoxic agent experiment are listed.

Title of gene set	Description
YEN MYC MUT′	Genes upregulated in mutant MYC mouse model
IGFR IR UP′	Upregulated in common following stimulation of chimeric TrkC IR or TrkC IGFR in NIH3T3 cells
3AB GAMMA DN′	Downregulated synergistically by gamma irradiation and 3-aminobenzamide PARP inhibitor
LEI HOXC8 UP	Upregulated target genes of murine transcription factor Hoxc-8
CHESLER D6MIT150 CIS GLOCUS′	Cis regulatory quantitative trait loci QTLs found at the D6Mit150 region QTLs detected in brain tissue
ROS MOUSE AORTA UP′	Upregulated in mouse aorta by chronic treatment with PPAR-gamma agonist rosiglitazone
YEN MYC WT′	Genes upregulated in wild type MYC mouse model
TNFALPHA ADIP UP′	Upregulated in mature differentiated adipocytes following treatment with TNFalpha
